# COVID-19 Serology Control Panel Using the Dried-Tube Specimen Method

**DOI:** 10.4269/ajtmh.21-1036

**Published:** 2022-01-07

**Authors:** William J. Windsor, Vijaya Knight, Patricia A. Merkel, Molly M. Lamb, Heidi R. Tucker, Kyle Carson, Kelly M. Howard, Jennifer L. Yates, Mario L. Santiago, Mary K. McCarthy, Thomas E. Morrison, Ross M. Kedl, Ashley Frazer-Abel, Kejun Guo, Gillian Andersen, Leah Huey, Bradley S. Barrett, Jessica M. Colón-Franco, William T. Lee, May C. Chu

**Affiliations:** ^1^ Colorado School of Public Health, Anschutz Medical Campus, Aurora, Colorado;; ^2^ Children’s Hospital Colorado, Anschutz Medical Campus, Aurora, Colorado;; ^3^ Division of Infectious Diseases, Wadsworth Center, New York State Department of Health, Albany, New York;; ^4^ The School of Public Health, The University at Albany, Albany, New York;; ^5^ University of Colorado Anschutz Medical Campus, Aurora, Colorado;; ^6^ Cleveland Clinic, Cleveland, Ohio

## Abstract

The dried-tube specimen (DTS) procedure was used to develop the COVID-19 serology control panel (CSCP). The DTS offers the benefit of shipping materials without a cold chain, allowing for greater access without deterioration of material integrity. Samples in the panel were sourced from COVID-19 convalescent persons from March to May 2020. The immunoglobulin subtypes (total Ig, IgM, and IgG) and their respective reactivity to severe acute respiratory syndrome coronavirus 2 nucleocapsid, spike, and receptor-binding domain antigens of the samples were delineated and compared with the WHO International Standard to elucidate the exact binding antibody units of each CSCP sample and ensure the CSCP provides adequate reactivity for different types of serological test platforms. We distribute the CSCP as a kit with five coded tubes to laboratories around the world to be used to compare test kits for external quality assurance, for harmonizing laboratory testing, and for use as training materials for laboratory workers.

A standardized panel composed of well-characterized plasma/serum specimens can bridge serosurveillance studies, compare test kits, serve as external quality assurance, harmonize tests that measure vaccine efficacy, be used as a training tool for laboratory workers, and be used for post-market monitoring. The standardized panel can be shared by a global network to link studies and enable inclusive analysis for a variety of use cases, as mentioned, and, more importantly, a standardized control panel can provide long-term quality performance monitoring as reagents and production batches change. We have established a COVID-19 serology control panel (CSCP) using the dried-tube specimen (DTS) protocol^1^ so that the panel can be shipped globally without a cold chain, thus allowing greater access to materials in all resource settings while maintaining sample integrity. Identifying the appropriate test kit for a use case has been made more complicated with more than 120 severe acute respiratory syndrome coronavirus 2 (SARS-CoV-2) serological test kits listed by the U.S. Food and Drug Administration under Emergency Use Authorized and/or registered with the Conformitè Européenne-marked European market. The SeroTracker^2^ list shows there are as many research use-only tests being used in clinics and research laboratories. With this unprecedented number of serological testing platforms and algorithms, it is imperative that we prioritize the quality calibration of test kits and platforms to ensure results are meaningful and can be compared across the hundreds of seroprevalence studies being undertaken.^3^ Calibration is especially important for testing in low-resource settings, where immunological testing is more likely to be used than other diagnostic test formats.

Nine highly reactive COVID-19 convalescent plasma samples collected between March and May 2020 by the Vitalant Research Institute (San Francisco, CA) were selected. The selected samples had a neutralization reactivity range of 1:640 to > 1:10,240 using a SARS-CoV-2 spike (S) reporter viral particle neutralization assay, and reactivity was confirmed using the Ortho VITROS Anti-SARS-CoV-2 Total immunoglobulin assay (S subunit protein; Ortho-Clinical Diagnostics, Inc. Rochester, NY) (Table 1).^4^ These nine COVID-19 convalescent plasma samples and one pre-2019 human plasma sample were certified to be blood borne and pathogen free by the Vitalant Research Institute. This study was conducted under a University of Colorado–Denver (CU) Human Subjects Research Waiver (protocol 20-0711).

**Table 1:** Test platforms used to characterize the COVID-19 serology control panel samples

Antigens	Antibody type	Format	Test output	Source	Details
Individual donor plasma characterization					
S1	Total Ig	Chemiluminescent immunoassay	Qualitative	Ortho Vitros Cov2T	EUA
	Total Ig	Pseudo-type VSV reporter neutralization	Quantitative	VRI	RUO; results reported as NT50
Individual and pooled donor plasma characterization					
S1	Total Ig	Pseudo-type HIV reporter neutralization	Quantitative	CU Anschutz	RUO; results reports as NT
RBD and N	IgG	ELISA	Qualitative	CU Anschutz	RUO
	IgG	Multiplex microsphere immunoassay	Quantitative	CU Anschutz	RUO
N	IgM and IgG	ELISA	Qualitative	Epitope	EUA
Virus	Total Ig	Focus reduction neutralization titer	Quantitative	CU Anschutz	Vero E6 cells cultured with SARS-CoV-2 USA-WA1/2020 strain

CU = University of Colorado; EUA = U.S. Food and Drug Administration Emergency Use Authorization; N = nucleocapsid proteins; NT = neutralization test; NT50 = neutralization test reported as 50% of reduction of virus replication; RBD = receptor binding protein; RUO = research use only; S1 = severe acute respiratory syndrome coronavirus 2 spike 1; SARS-CoV-2 = severe acute respiratory syndrome coronavirus 2; VRI = Vitalant Research Institute; VSV = vesicular stomatitis virus.

The samples were evaluated by CU Laboratories to determine each sample’s reactivity to the S, nucleocapsid (N), and receptor-binding domain (RBD) with five SARS-CoV-2 serology methods (Table 1).^5^^–^^8^ We pooled three samples that represented the highest reactivity to S, N, and RBD in a 1:1:1 ratio. The undiluted pool served as the high-reactive (HR) sample and the pre-2019 plasma served as the non-reactive (NR) sample in all assays. The low-reactive (LR) pool was prepared as a 1:4 dilution of the HR using the NR sample as the diluent. We included duplicate LR samples in the CSCP to provide insights into serology assay limits of detection. Then, the three samples—HR, LR, and NR—were evaluated by CU Laboratories pre- and post-drying.

According to the DTS protocol,^1^ we mixed in 0.1% green food dye for better visualization. We aliquoted 20 μL in a 2-mL Sarstedt vial (NC9180825; Fisher Scientific, USA) and then left the open tube to dry overnight in a high-efficiency particulate air-filtered laminar flow hood. The tubes were capped and stored at 4°C during the CSCP kit assembly process, and stored at –20°C afterward to preserve the integrity of the samples for longer term storage. To rehydrate, a DTS vial was rehydrated with 200 μL phosphate-buffered saline (PBS) with 0.2% Tween (PBS diluent), then was allowed to solubilize overnight at 4°C before use.

The long-term temperature stability of the dried materials was determined by storing CSCP kits continuously at –20°C, 4°C, 25°C, 37°C, and 45°C for 1 week, 2 weeks, 1 month, 3 months, 6 months, and 1 year. The optimal stability of the CSCP for up to 1 year is between –20°C and 25°C, with loss of reactivity after 2 months at 37°C and non-reactivity at 45°C (data not shown).

The CSCP was further characterized and measured against the WHO International Standard (WHO IS).^10^ We used the microbead immunoassay^9^ for this characterization, and analyzed results by parallel line assay (PLA). The immunoassay reagent preparation protocol and the derivation of the PLA analysis are provided in the Supplemental Materials.

PLA is the standard approach used to convert any analyte to binding antibody units (BAUs) against a known concentration of an analyte standard, represented here as the WHO IS N, S, and RBD-specific Ig. IgM and IgG were chosen as the analytes to convert HR and LR samples to BAUs. The WHO IS was set to 1,000 BAU/mL for each antigen–isotype combination, and the CSCP DTS HR and LR samples were considered unknowns. A robust PLA is dependent on the linearity of the dilution curves for both the standard and unknown. Therefore, the dilution curves of all analytes were linearized using a logit transformation on the raw data for both the CSCP DTS standard and WHO IS, measured as median fluorescence intensity. The logit transformation resulted in six to nine serial dilutions within the parallel linear range for comparison of all analytes except N-specific IgM (Figure 1). The CIs for these calculations were less than 25% (Table 2). However, we were unable to predict a potency for N-specific IgM because of a large difference between the slopes of the WHO IS and CSCP CTS standard for this analyte. Although detectable, the levels of N-specific IgM were extremely low in both the WHO IS and CSCP HR and LR samples, which is the likely cause for the low predictive value of this analyte.^10^^,^^11^

**Figure 1.**
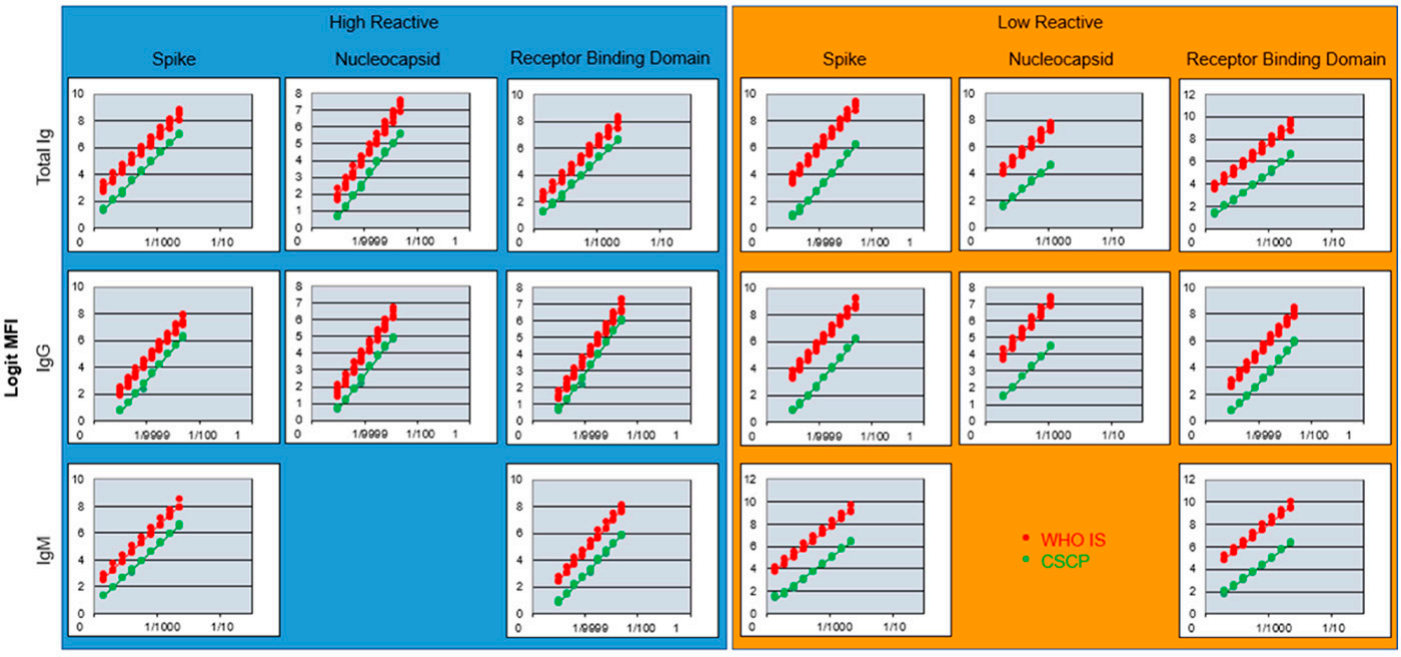
Parallel line assay results of COVID-19 serology control panels (CSCPs) compared with the WHO International Standard (WHO IS). Parallel lines obtained by plotting logit-transformed median fluorescence intensity (MFI) for the dilution ranges used to calculate binding antibody units per milliliter of total antibody (Ig), IgG, and IgM against the monomeric full-length spike, nucleocapsid, and receptor-binding domain of severe acute respiratory syndrome coronavirus 2 in the high-reactive and low-reactive CSCP reconstituted dried-tube specimen. The WHO severe acute respiratory syndrome coronavirus 2 serology international standard is in red and the CSCP specimen is in green. This figure appears in color at www.ajtmh.org.

**Table 2:** Potency of COVID-19 serology control panel standards in international binding antibody units

	High-reactive sample	Low-reactive sample 1	Low-reactive sample 2
Ig			
* *Nucleocapsid proteins	212.1 (185.3–241.9)*	49.7 (38.7–62.5)	58.4 (47.7–70.5)
* *Spike	215.8 (190.0–244.3)	50.5 (43.2–58.6)	58.0 (49.9–67.1)
* *Receptor-binding domain	295.1 (260.3–333.6)	72.1 (62.0–83.3)	81.3 (70.2–93.6)
IgM			
* *Nucleocapsid proteins	n/a	n/a	n/a
* *Spike	231.8 (206.9–259.2)	54.7 (47.6–62.5)	64.4 (56.2–73.4)
* *Receptor-binding domain	145.8 (129.5–163.6)	35.3 (29.7–41.6)	39.5 (33.5–46.2)
IgG			
* *Nucleocapsid proteins	257.7 (222.6–297.0)	55.8 (43.4–70.0)	55.4 (44.4–68.1)
* *Spike	246.1 (215.9–279.7)	57.0 (48.8–66.2)	57.6 (49.2–67.1)
* *Receptor-binding domain	408.3 (364.0–457.3)	101.0 (88.4–114.8)	97.3 (85.1–110.8)

n/a = not applicable.

* Binding antibody units based on WHO severe acute respiratory syndrome coronavirus 2 serology international standard as determined by parallel line analysis ± CI (%).

A CSCP kit contains five DTS samples (blinded), 200 μL PBS diluent, a 0.5-mL calibrated disposable micropipette, a dry silica pack, a printed DTS rehydration work aid, and a copy of the report form. The contents are sealed in a 1.5- × 9-inch mailing tube. Users fill out a CSCP request form and each request is reviewed, assessed, and approved before the requested CSCP kit is shipped at ambient temperature with no cold packs. Users then use the kit according to their serology assay requirements. Users return their results and provide the information of the test platform used via electronic entry (CSCP Result Form) or e-mail a copy to COVIDPanel@ucdenver.edu. A report decoding their samples is returned to the user; the report provides a comparison and interpretation of their results against the assigned value for the DTS samples they received. We provide a root-cause analysis to assist users in analyzing and determining corrective measures should the results provided be discordant with the assigned values.

As of June 2021, the CSCP has been shipped to multiple sites in Australia, Africa, Southeast Asia, North and South America, and Europe. CSCP concordance of the HR, NR, and LR samples are 97%, 93%, and 65%, respectively. We anticipated the LR sample would be a measure of the sensitivity of the test kit because of its construction or read-out method of the test platform; we are collecting these data for further analysis. Users have experienced a variety of logistical challenges, including receiving kits that were in transit for up to 3 months under harsh conditions (*n *= 1) and kits that were held for months before use (*n *= 2) without change in the expected results. One user reported issues with incomplete reconstitution and two users had problems uploading results to the website, all of which are under review for corrective actions.

Quality assurance is foundational for the validation of methods, external quality assurance, training, and inter-/intra-laboratory comparison of serological tests.^3^^,^^12^^–^^14^ As global COVID-19 vaccination efforts are now underway, highly accurate and reliable SARS-CoV-2 serology testing is the primary method to assess vaccine efficacy.^15^ Many commercial and laboratory-developed tests react with a range of antigen targets, making it difficult to compare results in the absence of a common set of reference materials. To address this need, the CSCP was further evaluated against the WHO IS using a microsphere immunoassay capable of measuring IgM, IgG, and total Ig reactivity to the S, RBD, and N antigens. By converting the CSCP HR and LR samples to the WHO IS BAUs, we provide the opportunity for direct inter- and intra-laboratory comparison of SARS-CoV-2 serological test results using calibrated reference samples. Widespread use of the CSCP for comparison of SARS-CoV-2 tests will help laboratories interpret and gain confidence in their results, while deterring laboratories from using poorly performing tests. In addition, the CSCP will help clinical laboratories inform their choice of diagnostic test to supplement clinical diagnoses of SARS-CoV-2 infection.

With this use in mind, our next step is to harmonize CSCP and other available serology reference materials by validating them concomitantly as secondary standards to the WHO IS. This would provide an inferential link to WHO IS and give broader access of validated reference materials to be used in comparing and evaluating test kit performance in use cases already cited. The DTS system is also flexible enough to accommodate additional samples to reflect current pandemic situations, such as post-vaccination and convalescent samples from persons infected with SARS-CoV-2 variants.

## Supplemental Material


Supplemental materials

## References

[1] Parekh BS 2010. Dried tube specimens: a simple and cost-effective method for preparation of HIV proficiency testing panels and quality control materials for use in resource-limited settings. J Virol Methods 163: 295–300. 19878697 10.1016/j.jviromet.2009.10.013

[2] Arora RK 2021. SeroTracker: a global SARS-CoV-2 seroprevalence dashboard. Lancet Infect Dis 21: e75–e76. 32763195 10.1016/S1473-3099(20)30631-9 PMC7402646

[3] Badrick T Wienholt L Fone D Holzhauser D , 2020. The challenge of producing an EQA for the COVID-19 pandemic. Pract Lab Med 22: e00179. 33102672 10.1016/j.plabm.2020.e00179 PMC7568490

[4] DiGermanio CSG 2021. SARS-CoV2 antibody persistence in COVID-19 convalescent plasma donors. MedRxiv.

[5] Hasenkrug KJ 2021. Recovery from acute SARS-CoV-2 infection and development of anamnestic immune responses in T cell-depleted rhesus macaques. bioRxiv. 10.1128/mBio.01503-21 PMC8406331 34311582

[6] EDI Epitope Diagnostics Inc. , n.d. *EDI™ Novel Coronavirus COVID-19 ELISA Kits*. Available at: http://www.epitopediagnostics.com/covid-19-elisa. Accessed December 1, 2021.

[7] Sabourin KR 2021. Risk factors of SARS-CoV-2 antibodies in Arapahoe County first responders: the COVID-19 Arapahoe Serosurveillance Study (CASES) project. J Occup Environ Med 63: 191–198. 33298759 10.1097/JOM.0000000000002099 PMC7934329

[8] Schultz JS 2021. Development and validation of a multiplex microsphere immunoassay using dried blood spots for SARS-CoV-2 seroprevalence: application in first responders in Colorado, USA. J Clin Microbiol 59. 10.1128/JCM.00290-21 PMC8315929 33795412

[9] Yates JL 2021. Serological analysis reveals an imbalanced IgG subclass composition associated with COVID-19 disease severity. Cell Rep Med. 2: 100329. 34151306 10.1016/j.xcrm.2021.100329 PMC8205277

[10] National Institute for Biological Standards and Control, World Health Organization , 2020. *WHO International Standard: First WHO International Standard for Anti-SARS-CoV-2 Immunoglobulin (Human): Instructions for Use*. Available at: https://www.nibsc.org/documents/ifu/20-136.pdf. Accessed December 1, 2021.

[11] World Health Organization , 2020. *Establishment of the WHO International Standard and Reference Panel for Anti-SARS-CoV-2 Antibody*. Available at: https://cdn.who.int/media/docs/default-source/biologicals/ecbs/bs-2020-2403-sars-cov-2-ab-ik-17-nov-2020_4ef4fdae-e1ce-4ba7-b21a-d725c68b152b.pdf?sfvrsn=662b46ae_8&download=true. Accessed December 1, 2021.

[12] Klein K 2020. A global proficiency testing programme for Xpert(R) MTB/RIF using dried tube specimens, 2013–2015. Afr J Lab Med 9: 1167. 33354528 10.4102/ajlm.v9i1.1167 PMC7736691

[13] Benzaken AS 2014. External quality assurance with dried tube specimens (DTS) for point-of-care syphilis and HIV tests: experience in an indigenous populations screening programme in the Brazilian Amazon. Sex Transm Infect 90: 14–18. 24031029 10.1136/sextrans-2013-051181

[14] Beber AM Sabido M Vieira JM Bazzo ML Benzaken AS , 2015. External quality assessment in the voluntary counseling and testing centers in the Brazilian Amazon using dried tube specimens: results of an effectiveness evaluation. Rev Soc Bras Med Trop 48: 87–97. 10.1590/0037-8682-0106-2014 26061375

[15] West RGG Kobokovich A , 2021. *Variants, Vaccines and What They Mean for COVID-19 Testing*. Available at: https://www.jhsph.edu/covid-19/articles/variants-vaccines-and-what-they-mean-for-covid19-testing.html. Accessed December 1, 2021.

