# Supplementary material for: COVID-19 Serology Control Panel Using the Dried-Tube Specimen Method
Source: Am J Trop Med Hyg. 2022 Jan 7;106(2):562–5. doi: 10.4269/ajtmh.21-1036 (PMC8832945; doi:10.4269/ajtmh.21-1036)
Supplement: Supplementary file 1 [file tpmd211036.SD1.pdf]

Supplementary Methods

Microsphere Immunoassay

Recombinant SARS-CoV-2 nucleocapsid (N), full-length spike (FLS) (Native Antigen, Oxfordshire, UK) and receptor binding domain (RBD) (MassBiologics, Boston, MA) were covalently linked to fluorescent microsphere beads using 1-ethyl-3-(3-dimethylaminopropyl) carbodiimide hydrochloride (EDC) and N-hydroxysulfosuccinimide (sulfo-NHS) in the xMAP Antibody Coupling Kit (Luminex Corporation, Austin, TX). Reconstituted CSCP DTS standards were diluted to 1:50 through 1:102,400 with 12 doubling dilutions in PBN (phosphate buffered saline, pH 7.4;1 % BSA; 0.05% sodium azide) (Sigma-Aldrich, St. Louis, MO). SARS-CoV-2 reactivity was measured using a microsphere immunoassay as previously described (Yates et al). Briefly, DTS standard dilutions and antigen-conjugated microspheres (25µl at $5x10^4$ microspheres/mL) were mixed and incubated for 30 minutes at 37°C. Serum-bound microspheres were washed and incubated with 50µl phycoerythrin (PE)-conjugated secondary antibody specific for human total Ig, IgM, or IgG, respectively (Southern Biotech, Birmingham, AL). After washing and final resuspension in buffer, the samples were analyzed on a FlexMap 3D analyzer using xPONENT software, version 4.3 (Luminex Corporation).

Parallel Line Analysis

CSCP DTS standards were converted to binding antibody units (BAU) as determined by comparison with the World Health Organization international standard (WHO IS; set at 1000 BAU/mL for each analyte) by parallel line analysis (PLA). To characterize the WHO IS, each standard was tested in triplicate on four separate FlexMap 3D analyzers (Luminex Corporation) with the average of all runs used for the analysis. The CSCP DTS standards were also tested in triplicate with the average used in the PLA. Data was analyzed in a Microsoft Excel macro-Enabled worksheet modified from PLA Tredecim V01Sep2018 (Ed Nieuwenhuijs (2019). https://ednieuw.home.xs4all.nl/Calibration/PLA/. PLA tredecim V01Sep2018.xlsm; Manual_PLA_Tredecim_V01Sep2018_in_Excel) using Excel version 2102 in Microsoft 365 (Microsoft Corporation, Redmond, WA). The worksheet was modified to perform the logit transformation optimized to the WHO IS and the maximum number of dilutions included in the analysis was extended from 7 to 9 allowing for better linearization of measures at a higher concentration and therefore inclusion of additional data points; increasing the overall accuracy of final calculations. For the logit transformation, a Bmax was chosen that resulted in a correlation coefficient between the Ln(mean MFI WHO IS/(Bmax- mean MFI WHO IS)) and Ln(WHO IS dilution) that was closest to 1. The best-fit transformation was then applied to all MFIs for the WHO IS and CSCP standard. The minimum (lowest logit-transformed MFI) was subtracted to make all values positive.
